# Potential Use of Human Periapical Cyst-Mesenchymal Stem Cells (hPCy-MSCs) as a Novel Stem Cell Source for Regenerative Medicine Applications

**DOI:** 10.3389/fcell.2017.00103

**Published:** 2017-12-05

**Authors:** Marco Tatullo, Bruna Codispoti, Andrea Pacifici, Francesca Palmieri, Massimo Marrelli, Luciano Pacifici, Francesco Paduano

**Affiliations:** ^1^Stem Cells Unit, Biomedical Section, TECNOLOGICA S.r.l., Marrelli Health, Crotone, Italy; ^2^Department of Oral and Maxillofacial Sciences, Sapienza University of Rome, Rome, Italy

**Keywords:** mesenchymal stem cells (MSCs), human periapical cyst mesenchymal stem cells (hPCy-MSCs), cell therapy, waste medicine, regenerative medicine

## Abstract

Mesenchymal stem cells (MSCs) are attracting growing interest by the scientific community due to their huge regenerative potential. Thus, the plasticity of MSCs strongly suggests the utilization of these cells for regenerative medicine applications. The main issue about the clinical use of MSCs is related to the complex way to obtain them from healthy tissues; this topic has encouraged scientists to search for novel and more advantageous sources of these cells in easily accessible tissues. The oral cavity hosts several cell populations expressing mesenchymal stem cell like-features, furthermore, the access to oral and dental tissues is simple and isolation of cells is very efficient. Thus, oral-derived stem cells are highly attractive for clinical purposes. In this context, human periapical cyst mesenchymal stem cells (hPCy-MSCs) exhibit characteristics similar to other dental-derived MSCs, including their extensive proliferative potential, cell surface marker profile and the ability to differentiate into various cell types such as osteoblasts, adipocytes and neurons. Importantly, hPCy-MSCs are easily collected from the surgically removed periapical cysts; this reusing of biological waste guarantees a smart source of stem cells without any impact on the surrounding healthy tissues. In this review, we report the most interesting research topics related to hPCy-MSCs with a newsworthy discussion about the future insights. This newly discovered cell population exhibits interesting and valuable potentialities that could be of high impact in the future regenerative medicine applications.

## Introduction

Stem cell therapy has reached a central role in the regenerative medicine scenario with numerous examples of translational applications reported in clinical trials designed for the most various pathological conditions.

The first lineage of adult stem cells was discovered in bone marrow, such bone marrow-derived cells were hematopoietic progenitors capable to reconstitute the entire blood system in immunocompromised hosts (Till and Ea, 1961). A few years later, Friedenstein first described a population of “clonal, plastic-adherent cells” residing in the same anatomical place (Friedenstein et al., 1968). These newly discovered cells, similar to hematopoietic stem cells, were able to self-renew, and moreover possessed the ability to differentiate into cells of mesodermal origin such as adipocytes, osteoblasts and chondrocytes. This population was named “mesenchymal stem cells” (MSCs) and was phenotypically defined by the expression of specific surface markers such as CD105, CD90, CD73, and by the lacking of markers typically expressed by hematopoietic cells including HLA-DR, CD45, and CD34 (Barry and Murphy, 2004).

Bone marrow was initially considered the main source of MSCs. Afterwards, several authors demonstrated that MSCs are present in different human tissues such as blood, umbilical cord, placenta, fat, heart, brain, skin, muscle, liver, gonads, and teeth (Chamberlain et al., 2007; Egusa et al., 2012). Importantly, human umbilical cord (UC), placenta and adipose tissue are promising sources of mesenchymal stem cells. Umbilical cord mesenchymal stem cells (UC-MSCs) are easily obtained from UC without ethical implications and exhibit excellent multipotency (Van Pham et al., 2016). Human placental MSCs (PL-MSCs) display the capability of multipotential differentiation and retain a strong immunosuppressive potential (Chen et al., 2015). Adipose tissue is an optimal source of proliferating, non-immunogenic, and easily available stem cells (ASCs). This population presents an intense paracrine activity that is able to induce strong biological effects on surrounding cells such as the promotion of cell proliferation and differentiation, as well as the activation of reparative and regenerative mechanisms (Paduano et al., 2017b). Furthermore, several studies demonstrated that MSCs can differentiate toward several cell lineages and can contribute to regeneration/reparation of a wide range of the adult tissues including skeletal muscles, tendons, and neuronal cells, demonstrating that MSCs hold an amazing plasticity (Phinney and Isakova, 2005; Catacchio et al., 2013). The capability of MSCs to differentiate into several cell types, as well as their important immunomodulatory effects, make them an attractive therapeutic tool for regenerative medicine, including cell transplantation and tissue engineering.

Moreover, the immunomodulatory and restorative effects of MSCs have been exploited for regenerative therapy in oncologic patients after tumor ablative techniques. However, the paracrine pro-angiogenic, anti-apoptotic, and pro-survival properties of MSCs have been implicated by different studies as the possible triggering of cancer recurrence (Zimmerlin et al., 2013). Another study described the inhibition of tumor growth exerted by MSCs through the down-regulation of survival signaling such as Wnt and Akt pathways. It is known that MSCs have the ability to migrate and engraft into tumor sites, but the real effect on cancer growth/inhibition still remains a subject of debate (Hong et al., 2014).

Collection of MSCs from human bone marrow (hBM) does not imply a quite simple procedure, indeed, donors must undergo an invasive surgery to allow the proper aspiration of bone marrow from the iliac crests. Moreover, cells obtained from hBM are in a limited number, estimated to be nearly of one MSC per 34,000 nucleated cells (Beyer Nardi and da Silva Meirelles, 2006). These issues have attracted the interest of researchers that are aiming to search for alternative sources of MSCs that can be obtained without the use of invasive and painful procedures.

## Oral derived stem cells

In the last years, several research groups have carefully analyzed the oral and dental tissues as potential sources of MSCs. Oral-derived stem cells (ODSCs) can be easily isolated from fresh teeth extracted because of orthodontic or periodontal reasons. The great accessibility to the oral tissues and the high abundance of MSC-like cells in such anatomical district (Tatullo et al., 2015c) have led ODSCs to be one the most studied type of MSCs.

During the last decades, it has been commonly hypothesized that teeth develop their structure from two different embryonic tissues: ectodermal epithelium and ectomesenchymal neural crests, giving rise to specific tissues like pulp and dentine (Koussoulakou et al., 2009). Using a mouse model of tooth organogenesis, Kaukua et al. showed that a significant MSCs population, highly active in dental tissue repairing, were derived from peripheral nerve-associated glia. Thus, supporting the idea that pulp cells and odontoblasts are generated from multipotent mesenchymal progenitors originated by glial cells (Kaukua et al., 2014).

**Figure 1:**
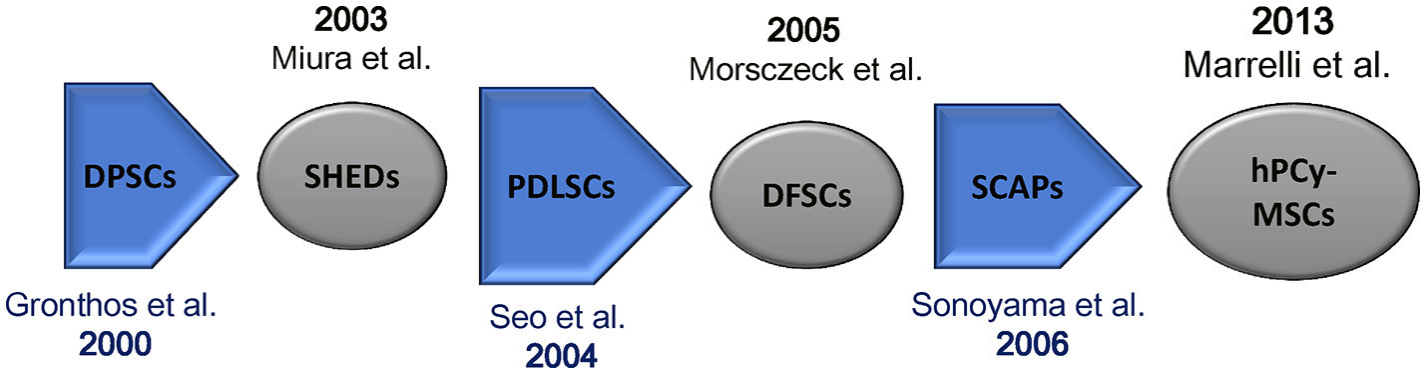
Mesenchymal stem cells isolated from oral tissues. Chronologic flow. DPSCs, dental pulp stem cells; SHEDs, stem cells from human exfoliated deciduous teeth; PDLSCs, periodontal ligament stem cells; SCAPs, stem cells from apical papilla; hPCy-MSCs, human periapical cyst-mesenchymal stem cells.

In 2000, Gronthos et al. first described the existence of cells in adult human dental pulp that are capable of self-renewal and called these cells as dental pulp stem cells (DPSCs). Such cells were also able to differentiate toward dentin/pulp-like tissues after transplantation into immunocompromised mice. Importantly, DPSCs possess the same immunophenotype of bone marrow stromal cells (BMSCs) (Gronthos et al., 2000; Figure 1). DPSCs have been largely investigated; they are adult stem cells showing an optimal plasticity, indeed, they are able to differentiate into the three mesenchymal cell lineages (osteocytes, chondrocytes, and adipocytes) as well as into hepatocytes (Ishkitiev et al., 2010), myocytes (Yang et al., 2010), neurons (Iohara et al., 2006), and hair follicle cells (Reynolds and Jahoda, 2004).

Few years after the discovery of DPSCs, Miura and colleagues isolated multipotent, clonogenic, and highly proliferating cells from human exfoliated deciduous teeth (SHEDs); these cells showed the ability to differentiate into various cell types, also promoting the formation of dentin and bone tissues *in vivo* (Miura et al., 2003).

Moreover, a cell population expressing mesenchymal stem cell-like markers was found in periodontal ligament (PDL). Periodontal ligament stem cells (PDLSCs) can differentiate into adipocytes, collagen-forming cells, and cementoblast-like cells. Importantly, these cells have been successfully used to regenerate both cementum and PDL in animal models (Seo et al., 2004).

Developing teeth are covered by a thick connective tissue, defined as dental follicle, containing MSCs called dental follicle progenitor cells (DFPCs). These cells were first isolated by Morsczeck et al. (2005) in the literature, such cells have been described to promote neural regeneration as well as to regenerate periodontal and bone tissues (Morsczeck et al., 2005). Moreover, also root apical papilla of human teeth was found to be rich in stem cells. These cells known as stem cells from apical papilla (SCAP) can differentiate into osteogenic and odontogenic progenitors (Sonoyama et al., 2006; Yang et al., 2017).

Gingival tissues have been shown to be colonized by multipotent clonogenic stem and progenitor cells. Gingiva-derived MSCs have a clear regenerative potential comparable to BMSCs (Tomar et al., 2010). Moreover, also the periosteum covering the jawbones was identified as a source of MSC-like cells able to differentiate into chondrocytes and osteoblasts (Hutmacher and Sittinger, 2003).

Few researches have been also focused on human parotid glands: surprisingly, such tissues contain cells expressing both epithelial and mesenchymal specific markers and these cells are able to form colonies, under specific culturing conditions (Yi et al., 2016).

In 2013, Marrelli et al. discovered a new source of MSCs of dental origin; they demonstrated for the first time the presence of MSCs in human periapical cysts, which were termed human periapical cyst-mesenchymal stem cells (hPCy-MSCs) (Marrelli et al., 2013).

## Human periapical cyst-mesenchymal stem cells

Endodontic infections could lead to the formation of fibrous inflammatory tissues, richly infiltrated by macrophages, neutrophils and lymphocytes in the periapical area, with a consequent onset of apical periodontitis; the chronicization of such inflammatory condition may evolve into periapical cysts formation (Nair, 2004).

Clinical observations showed the formation of new bone in that periosteum area after surgical removal of periapical cyst, suggesting that stem cells could be involved in the regenerative process. A previous work reported by Maeda introduced the hypothetical occurrence of osteogenic cells in periapical granulation tissue (Maeda et al., 2004). After this first study, Patel and Liao described the presence of mesenchymal stem cell-like cells in granulation tissue, characterized by an intense osteogenic commitment (Patel et al., 2010; Liao et al., 2011).

In the light of these preliminary studies, periapical cysts were further investigated for the presence of MSCs: Marrelli et al. isolated and fully characterized a new cell population named hPCy-MSCs that could be considered one of the most promising MSCs in the tissue regeneration landscape.

## hPCy-MSCs: isolation and characterization

hPCy-MSCs isolation starts with a mechanical disruption of the cystic wall obtained after surgery, with a sterile scalpel in phosphate-buffered saline (PBS) solution containing antibiotics. Then, periapical cystic tissue samples can be minced into small pieces and subjected to enzymatic digestion with type-I collagenase and dispase. Subsequently, samples can be filtered and seeded in culture medium added with fetal bovine serum (FBS) (Huang et al., 2006). Freshly isolated hPCy-MSCs have a fibroblast-like morphology and can be plated up to 20 passages without losing their characteristics. Importantly, hPCy-MSCs possess stem-cell-like properties, including extensive proliferative potential, self-renewal capacity, and multi-lineage differentiation ability (Marrelli et al., 2013).

Freshly collected hPCy-MSCs, similar to other types of dental-derived MSCs such as DPSCs, DFPCs, and PDLSCs (Table 1), highly express CD13, CD29, CD44, CD73, CD90, CD105, STRO-1, and CD146 with a physiological variability among samples; in addition, these cells do not express hematopoietic markers, such as CD45 (Paduano et al., 2016a). Paduano et al. demonstrated the central role of the cell adhesion receptor CD146 in influencing hPCy-MSCs properties. In this work, the authors isolated two populations expressing low and high levels of CD146 and demonstrated how the CD146^low^ population displayed an increased proliferative and clonogenic potential, showing increased levels of the stemness gene marker *KLF4*; furthermore, CD146^low^ population had higher osteogenic potential compared to CD146^high^ cells (Paduano et al., 2016a).

**Table 1:** Comparative analysis of the expression of MSC-surface markers in four cell types of dental-derived MSCs.

	**hPCy-MSCs**	**DPSCs**	**DFPCs**	**PDLSCs**
CD13	+++	+++	+++	+++
CD29	+++	+++	+++	+++
CD44	+++	+++	+++	+++
CD73	+++	+++	+++	+++
CD45	–	–	–	–
CD90	+++	+++	+++	+++
CD105	+++	+++	+++	+++
CD146	++	++	+	+++

*Flow cytometry analysis of common MSC markers. Expression pattern of MSC markers in hPCy-MSCs, human periapical cyst-mesenchymal stem cells; DPSCs, dental pulp stem cells; DFPCs, dental follicle stem cells; PDLSCs, periodontal ligament stem cells at early passages after isolation. +++, ++, +, and – indicate high, moderate, low, and absent expression of MSC surface marker, respectively (Paduano et al., 2016a)*.

## Lineage commitments of hPCy-MSCs

hPCy-MSCs possess the classic trilineage differentiation potential into osteogenic, adipogenic, and chondrogenic lineages (Marrelli et al., 2013).

hPCy-MSCs after about 3 weeks of culturing in osteogenic medium, showed the formation of calcium-based nodules intensely positive to alizarin red staining, indicating the osteogenic differentiation potential of hPCy-MSCs (Marrelli et al., 2013). In addition, the mRNA levels of bone specific-genes, such as osteopontin (*OPN*), osteocalcin (*OSC*), alkaline phosphatase, and dentin matrix protein 1 (*DMP-1*) increased after osteogenic induction (Marrelli et al., 2013). Importantly, the same authors observed that, when compared to DPSCs under the same conditions, hPCy-MSCs are preferably directed toward osteogenesis whereas DPSCs were preferentially oriented toward dentinogenesis (Tatullo et al., 2015a). qRT-PCR analyses further revealed differences in the gene expression pattern among these two populations. The expression levels of dentin sialophosphoprotein (*DSPP*) and *DMP-1* genes were increased in DPSCs with respect to hPCy-MSCs, whereas osteonectin (*ON*), bone sialoprotein (*BSP*), and runt-related transcription factor 2 (*RUNX-2*) were highly expressed in hPCy-MSCs compared to DPSCs (Tatullo et al., 2015a). *In vivo* studies already confirmed the formation of mineralized tissues after subcutaneous transplantation of periapical granulation tissue derived-cells into NOD.CB17-Prkdc^scid^/J mice (Liao et al., 2011).

Furthermore, hPCy-MSCs seeded in adipogenic media can form cytosolic lipid droplets that positively stain to oil red, indicating the adipogenic differentiation potential of hPCy-MSCs. qPCR investigations confirmed the augmented expression of adipose-specific genes including: lipoprotein lipase (*LPL*), adiponectin (*ADIPOQ*), peroxisome proliferator-activated receptor gamma (*PPAR*γ), and glucose transporter type 4 (*GLUT4*) (Marrelli et al., 2013; Table 2).

**Table 2:** Comparative expression of differentiation markers in dental pulp stem cells and hPCy-MSCs.

	**Osteogenic**	**Adipogenic**	**Neurogenic**	**References**
DPSCs	OSC, ON, OPN, BSP, DSPP, RUNX-2, DMP-1	PPARγ, LPL	GFAP, β-III tubulin, NF-H, NF-M, Nestin, MAP2, NSE, MSX-1, Pitx3, Foxa2, Nurr1, EN1, TH, DAT	Gronthos et al., 2002; Marrelli et al., 2015b; Tatullo et al., 2015a
hPCy-MSCs	OSC, ON, OPN, BSP, DSPP, RUNX-2, DMP-1	GLUT4, LPL, ADIPOQ, PPARγ	GFAP, β-III tubulin, NF-H, NF-M, Nestin, MAP2, NSE, MSX-1, Pitx3, Foxa2, Nurr1, EN1, TH, DAT	Marrelli et al., 2013, 2015b; Tatullo et al., 2015a

*OSC, Osteocalcin; ON, osteonectin; OPN, osteopontin; BSP, bone sialoprotein; DSPP, dentin sialophosphoprotein; RUNX-2, runt-related transcription factor 2; DMP-1, dentin matrix protein 1; GLUT4, glucose transporter type 4; LPL, lipoprotein lipase; ADIPOQ, adiponectin; PPARγ, peroxisome proliferator-activated receptor gamma; LPL, lipoprotein lipase; NF-H, neurofilaments heavy; NF-M, neurofilaments medium; NSE, neuronspecific enolase; MSX1, Msh homeobox 1; MAP2, microtubule associated protein 2; GFAP, glial fibrillary acidic protein; PITX3, pituitary homeobox 3; NURR1, nuclear receptor related 1 protein; Foxa2, forkhead Box A2; EN1, engrailed homeobox 1; TH, tyrosine hydroxylase; DAT, dopamine transporter*.

To further confirm the multi-lineage differentiation capacity of these cells, the same author showed that hPCys-MSCs were able to effectively differentiate into neurogenic-like cells as revealed by the immunofluorescence, western blotting, and flow cytometry assays. Interestingly, they observed that the basal expression levels of neuronal and astrocyte-specific proteins (β-III tubulin and GFAP) in hPCys-MSCs were similar to those of DPSCs. Moreover, qRT-PCR analysis under basal culturing condition revealed higher levels of transcripts for neural-related genes (β-III tubulin, microtubule associated protein 2), neural-related transcription factors (msh homeobox 1, forkhead box protein A2, engrailed homeobox 1) and for dopamine-related genes (tyrosine hydroxylase and dopamine transporter) in hPCy-MSCs than in DPSCs. Importantly, the expression of neuronal markers were greatly increased under neurogenic conditions (neural medium containing retinoic acid, epidermal growth factor, and basic fibroblast growth factor). Thus, hPCy-MSCs cultured under neurogenic medium are able to acquire the typical neuron-like morphology, characterized by the presence of dendrite and axonal structures (Marrelli et al., 2015b). Taken together, these results showed that hPCy-MSCs possess a multi-lineage differentiation potential similar to that of other dental-derived MSCs such as DPSCs (Table 2).

## Potential clinical applications of hPCy-MSCs

Mesenchymal stem cells play a key role in regenerative medicine; after their discovery, MSCs were mainly collected from bone marrow or adipose tissue, however, the surgical procedures needed to collect such cells were invasive and often painful for patients. Therefore, the scientific community is actively involved in the discovering of alternative sources of MSCs. The oral cavity is strongly populated by MSCs that could be easily isolated in a minimally invasive way (Tatullo et al., 2015b). Thanks to their plasticity, oral derived MSCs can differentiate toward a wide range of specialized tissues, including fat, bone, cartilage, muscle, tendon, and neural tissues (Rosenbaum et al., 2008). In addition, oral derived MSCs produce anti-inflammatory and trophic effects by secreting several immunomodulatory molecules, supporting self-regulated healing processes in damaged tissues and providing a regenerative microenvironment (Figueroa et al., 2012).

ODSCs attracted clinical interest first in the oral and maxillofacial fields, as they have been recognized to be strategic for a better regeneration of bone and dental tissues, such as cementum, dentin and periodontal ligament. Many studies supported this concept: Seo et al. showed the ability of ODSCs to produce cementum and periodontal ligament after transplantation of human PDLSCs into immunocompromised mice (Seo et al., 2004). Zhang et al. seeded DPSCs on different scaffolds made of several materials including titanium, collagen, and sintered ceramic and investigated cell proliferation and differentiation on these scaffolds. As results, DPSCs were able to grow onto different scaffolds, moreover, they differentiated into osteocytes capable to generate a mineralized matrix (Zhang et al., 2012).

Nanoporous and mesoporous silicon scaffolds efficiently support DPSCs proliferation, thus they could represent a fine substrate for these cells in tissue engineering procedures (Marrelli et al., 2015a).

In the field of bone regeneration, hydrogel-based scaffolds can be obtained from decellularized and demineralized bovine bone extracellular matrix. Recent studies emphasized how ODSCs could be easily and efficiently cultured on hydrogels (Cavalcanti et al., 2013; Coyac et al., 2013) moreover, dental pulp stem cells are able to spontaneously differentiate toward both odontogenic (Paduano et al., 2016b) and osteogenic phenotype (Paduano et al., 2017a) when cultured on these scaffolds.

hPCy-MSCs have been reported to retain high proliferative rate and extensive multipotency. These key properties play a strategic role in bone and dental regeneration, even more if hPCy-MSCs are added to specific scaffolds. Furthermore, the recently discovered neural plasticity of these cells undoubtedly represents a perspective feature to be further investigated for future innovative strategies in brain repairing. In this light, stem cell therapies for some widespread neurodegenerative diseases, such as Alzheimer and Parkinson syndromes encountered many ethical issues related to the use of embryonic stem cells (Lees et al., 2009). Thus, many authors proposed alternative treatments based on adult mesenchymal stem cells to regenerate injured neural tissues (Uccelli et al., 2011).

**Figure 2:**
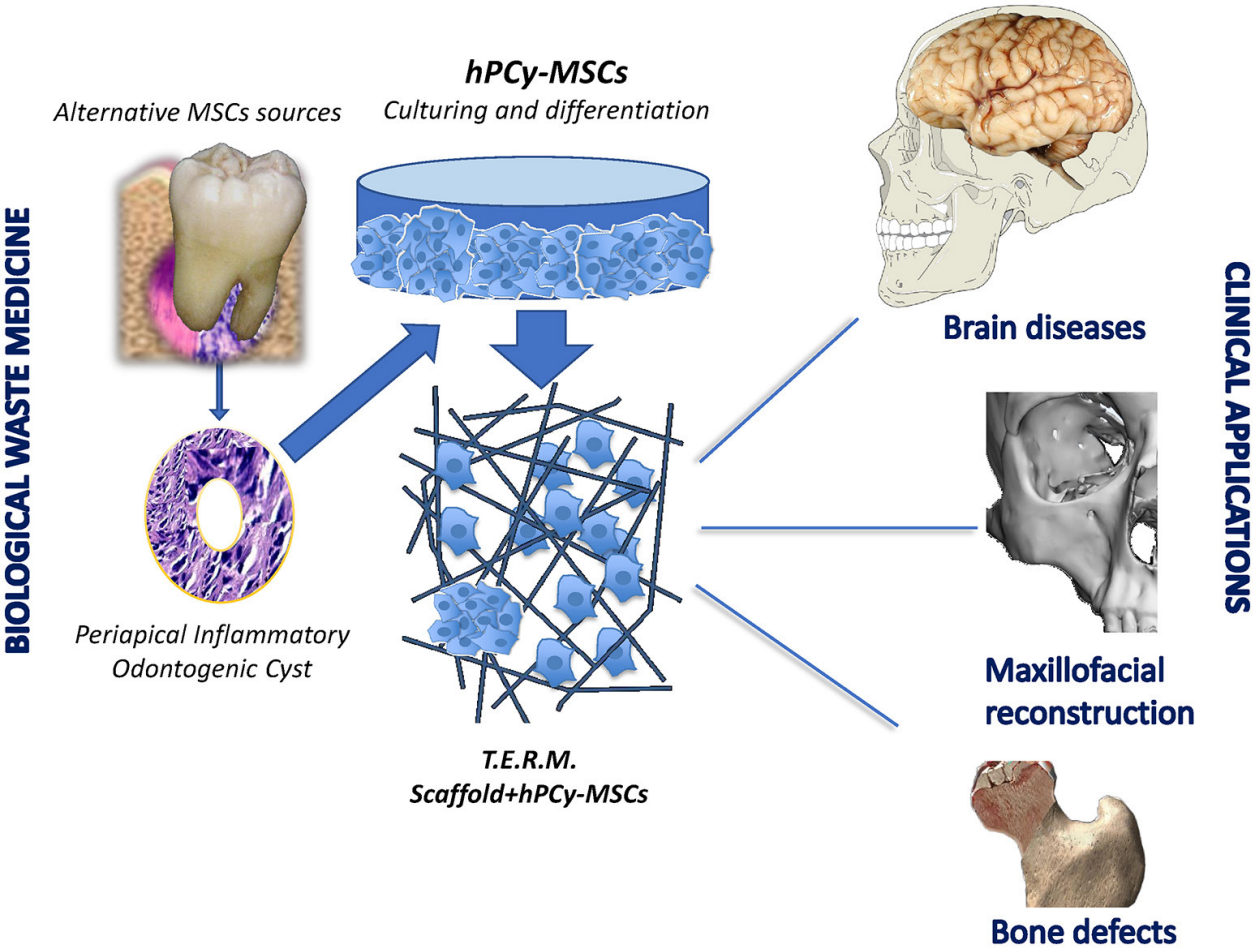
Clinical potentialities of hPCy-MSCs.

In the last few years, researchers are carrying out pioneering studies aimed to investigate the biochemical pathways in cellular models of Parkinson's disease. In this context, the spontaneous commitment of hPCy-MSCs to differentiate toward neuron-like cells arouses exciting insights about new therapies involving hPCy-MSCs. Further investigations are needed to evaluate the neuroregenerative potential of these newly discovered ODSCs in animal models (Figure 2).

## Conclusions

Dental periapical cysts are certainly among the most common lesions discovered after the observation of orthopanoramic radiographies; these benign formations are surgically eradicated by dentists to prevent the development of more severe pathologies, especially to the surrounding bone tissue.

Although inflamed cysts are routinely discarded as biological waste, recent researches revealed a strategic role of this granulation tissue (Liao et al., 2011), which represents a rich source of stem cells obtainable without any biological cost for patients.

The hypothesis reporting that biological wastes can be considered as resources for new unexpected biological applications have been largely discussed in the scientific literature, even in areas far from human biology, but closer to food biotechnology. For example, a recent study reported the extraction of bioactive compounds from food waste for pharmaceutical and medical purposes (Tatullo et al., 2016). Thus, many research topics perfectly fit with the need to use economic sources for medical applications. Periapical cysts fully reflect the modern concept of biological waste medicine, where rejected surgical tissues are exploited as precious fonts for novel medical applications (Tatullo, 2017).

Sources of abundant and highly immature MSCs, obtained from easily available and “biologically cheap” tissues, undoubtedly represent an extremely interesting and important finding. In this context, hPCy-MSCs, similarly to DPSCs, could be considered as an optimal cell source for tissue engineering applications. The use of human hPCy-MSCs is not free of criticisms since the immunomodulatory properties of these cells are not yet evaluated and most results were obtained from *in vitro* experiments. Thus, the real challenge now is to translate the *in vitro* results to an *in vivo* context, and therefore future investigation should be carried out to confirm the *in vivo* regenerative potential of these newly discovered MSCs.

## Author contributions

MT, FPad, MM, and BC: conceived and wrote the manuscript; BC, FPal, AP, and LP: generated the figures and revised the literature sources. All authors reviewed and approved the final manuscript.

## Conflict of interest statement

The authors declare that the research was conducted in the absence of any commercial or financial relationships that could be construed as a potential conflict of interest.
